# Persistent Cross-Institutional Domain Shift in Narrow-Band Imaging Classification of Colorectal Lesions and the Utility of Site-Specific Fine-Tuning

**DOI:** 10.3390/cancers18183002

**Published:** 2026-09-16

**Authors:** Hiroki Nomiya, Hidezumi Kikuchi, Takeshi Shimizu, Saki Nakano, Taka Asari, Yohei Sawada, Shohei Igarashi, Naoki Higuchi, Tetsuya Tatsuta, Daisuke Chinda, Yoshihiro Sasaki, Hirotake Sakuraba

**Affiliations:** 1 Department of Gastroenterology, Hematology, and Clinical Immunology, Hirosaki University Graduate School of Medicine, 5 Zaifucho, Hirosaki 036-8562, Japan; 2 Division of Endoscopy, Hirosaki University Hospital, 53 Honcho, Hirosaki 036-8563, Japan; 3 Department of Gastroenterology, Sendai City Medical Center, Sendai Open Hospital, 5-22-1 Tsurugaya, Miyagino-Ku, Sendai 983-0824, Japan; 4 Department of Gastroenterology, Misawa City Hospital, 164-65 Horiguchi, Misawa 033-0022, Japan; 5 Department of Community Medicine, Hirosaki University Graduate School of Medicine, 5 Zaifucho, Hirosaki 036-8562, Japan

**Keywords:** deep learning, colorectal lesion classification, narrow-band imaging, domain shift

## Abstract

**Background/Objectives:** Cross-institutional transfer of endoscopic deep learning models may be limited by acquisition and preprocessing differences. We reassessed persistent domain shift after correcting Facility B input linkage and validation design. **Methods:** A preflight audit corrected two Facility B image–mask linkage omissions without changing the 402 source lesions or 469 still images. Criterion B was a qualitatively selected, Facility-B-informed HSV patch-selection adjustment; the same Tenengrad focus criterion was used at both facilities. Class-wise MMD^2^ was calculated using all three Facility A fold-specific feature extractors. Each Facility A source model was evaluated on the same corrected criterion B Facility B cohort. Facility B models were initialized from ImageNet and fully fine-tuned for four prespecified epochs under lesion-grouped three-fold cross-validation, with pooled out-of-fold patch-level evaluation. Historical Facility A internal results were descriptive only. **Results:** Criterion B retained 142,878 Facility B patches. Residual MMD^2^ was greatest for SM1 (0.185) and SM2 (0.147). Cross-site accuracy, macro-F1, and balanced accuracy were 0.711, 0.397, and 0.422; site-specific values were 0.770, 0.472, and 0.473. Recall improved for BG, LG, and SM1 but decreased for HG and SM2. **Conclusions:** Site-specific development partially improved aggregate local performance, but persistent domain shift and class-specific limitations remained; universal cross-institutional generalizability was not established.

## Simple Summary

Deep learning models for colorectal lesion classification can lose accuracy when transferred between hospitals because image acquisition and data-processing distributions differ. We re-evaluated this problem using narrow-band imaging data from two facilities after an audit corrected two image–mask linkage omissions at Facility B without changing the source lesion or still-image cohort. A Facility-B-informed patch-selection rule increased the number of retained patches, yet feature distribution differences persisted, especially for invasive lesions, and Facility A models still showed substantial performance loss on the same corrected Facility B cohort. We therefore developed Facility-B-specific models using a validation design that kept all patches from the same lesion together. Aggregate patch-level performance improved, but recall improved for background, low-grade dysplasia, and superficially invasive cancer while decreasing for high-grade dysplasia and deeply invasive cancer. Historical Facility A internal results were not treated as directly comparable because the earlier validation split patches rather than lesions. These findings support site-specific model development as a pragmatic way to partially manage persistent domain shift, not as evidence of universal transferability.

## 1. Introduction

We previously developed a four-class histologic classification model utilizing narrow-band imaging (NBI) endoscopic images to differentiate low-grade dysplasia (LG), high-grade dysplasia (HG), superficially invasive submucosal carcinoma (SM1), and deeply invasive submucosal carcinoma (SM2). The model achieved high diagnostic performance, with patch-level and image-level accuracies ranging from 0.876 to 0.957 and from 0.983 to 0.992, respectively, even under non-magnified imaging conditions at Facility A (Sendai Open Hospital) [1]. Deep learning-based systems have also demonstrated high diagnostic performance for colorectal polyp characterization using NBI and other endoscopic imaging approaches [2,3,4,5,6]. However, their limitations included the lack of external validation and the unconfirmed generalizability of the model across institutions—a challenge well documented in medical image analysis and the clinical AI literature [7,8,9,10,11,12].

**Figure 1:**
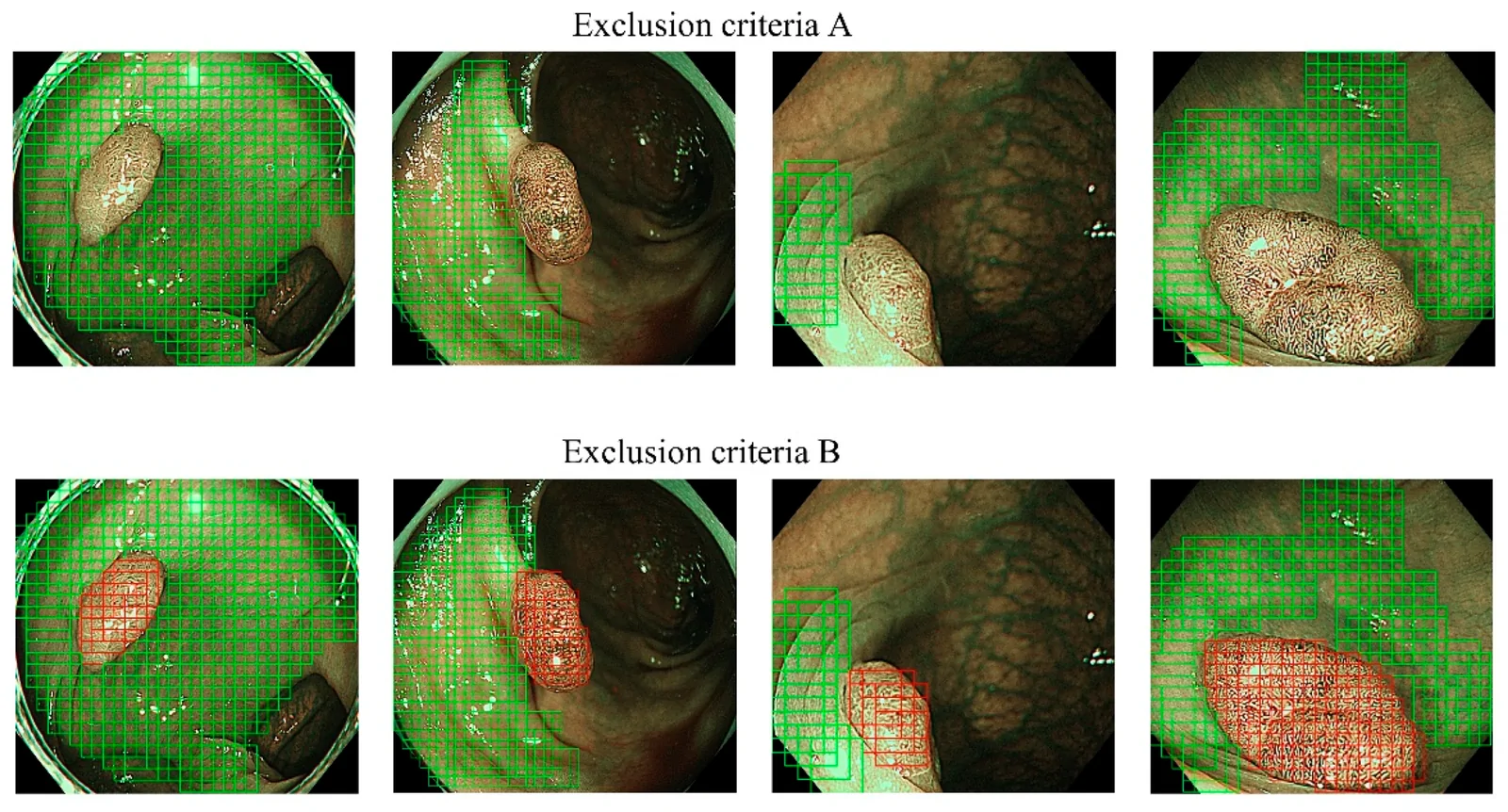
Comparison of patch selection results between exclusion criteria A and B. Each column represents an example case from Facility B. Green rectangles indicate background patches retained after cleansing based on the exclusion criteria. Red rectangles denote the retained lesion patches. The top and bottom rows represent exclusion criteria A and B, respectively.

To address this limitation, we conducted an external validation study using NBI images acquired at Facility B (Hirosaki University Hospital). The same patch exclusion criteria used in Facility A, targeting halation, blackout, and blurriness based on pixel intensity thresholds, were consistently applied. However, when these criteria were applied to images from Facility B, lesion patches were systematically excluded despite being clearly identifiable to experienced endoscopists (Figure 1, upper row). This discrepancy indicates a potential mismatch in lesion representation between the training and validation datasets, likely stemming from institution-specific image acquisition protocols. Changes in endoscopy system generations or endoscope models may likewise alter image characteristics and contribute to covariate shift and degradation of AI performance [11,13,14].

The primary aim of this study was to quantify persistent cross-institutional domain shift and its impact on patch-level classification performance after harmonizing patch selection criteria. A secondary aim was to evaluate whether site-specific full fine-tuning from ImageNet-pretrained weights could partially manage the local performance impact of limited model transferability. We did not aim to demonstrate elimination of domain shift or universal cross-institutional generalizability.

## 2. Methods

### 2.1. Ethical Considerations

This study was approved by the ethics committees of Hirosaki University School of Medicine (No. 2023-124) and Sendai City Medical Center (No. 2025-0099) and was conducted after obtaining informed consent in the form of an opt-out on our hospital website (https://www.med.hirosaki-u.ac.jp/hospital/outline/resarch.html (accessed on 13 September 2026)). It was also conducted in accordance with the Declaration of Helsinki.

### 2.2. Endoscopic Image Preparation

#### 2.2.1. Facility A

NBI images of neoplastic lesions from patients who underwent endoscopic or surgical resection at Sendai City Medical Center, Sendai Open Hospital, between April 2017 and December 2019 were used. The characteristics of the NBI images are summarized in Table 1A. The video endoscopes CF-HQ290ZI, PCF-H290ZI, and PCF-H290TI, as well as the video endoscopy system EVIS LUCERA ELITE CV-290/CLV-290SL (Olympus Medical Systems, Co., Ltd., Tokyo, Japan), were used for model training.

#### 2.2.2. Facility B

NBI images of neoplastic lesions from patients who underwent endoscopic or surgical resection at Hirosaki University Hospital between April 2017 and December 2019 were used for external validation. Characteristics of collected NBI images are summarized in Table 1B. The video endoscopes CF-HQ290ZI, PCF-H290ZI, and PCF-H290TI, as well as the video endoscopy system EVIS LUCERA ELITE CV-290/CLV-290SL (Olympus Medical Systems, Co., Ltd., Tokyo, Japan), were used.

### 2.3. Dataset Preparation

Patch-based datasets were constructed from the NBI images and lesion masks at both facilities. The final analysis retained the predefined patch-extraction settings while adding a preflight input-linkage audit and a lesion-grouped validation design for Facility B.

#### 2.3.1. Class Selection, Input Preparation, and Preflight Audit

Images were grouped into four lesion classes: LG, HG, SM1, and SM2. Corresponding lesion masks were available in grayscale format and were binarized using a threshold of 127. Before patch generation, each image–mask pair underwent a preflight check for mask availability, canonicalized filename linkage, image and mask readability, dimension matching, and the presence of positive lesion pixels. This audit identified two upstream linkage omissions in Facility B: one HG image whose mask had not entered the analysis pipeline, and one SM2 image whose mask failed filename matching because of a source-filename suffix. The mask linkage was corrected without changing the predefined patch extraction or quality exclusion criteria, and without changing the number of source lesions or still images.

#### 2.3.2. Patch Extraction Settings

The input images were divided into square patches of 128 × 128 pixels, with a stride of 32 pixels. Each patch was evaluated based on several exclusion criteria before inclusion in the training dataset.

#### 2.3.3. Patch Exclusion Criteria

Two patch exclusion criteria were evaluated using patch-level image quality measures. In our previous Facility A study [1], blackout regions were defined as pixels with a red-channel intensity < 50, and patches were excluded when blackout regions occupied >10% of the effective area. Halation regions were defined as pixels with a green-channel intensity > 250, and patches were excluded when halation regions occupied >5% of the effective area. Image sharpness in that study was assessed from spatial high-frequency components obtained using a high-pass filter with a cutoff frequency of 6.25% of the Nyquist frequency.

For the present study, the original blackout and halation definitions and thresholds were retained. The focus assessment procedure was operationally replaced by the Tenengrad measure for computational efficiency. A Tenengrad value < 5000 was used to identify insufficiently focused patches [15,16]. This replacement did not alter the source lesion or still-image cohort at Facility A; it only changed the operational focus quality criterion used for patch eligibility in the present study. The same Tenengrad criterion was applied consistently to both Facility A and Facility B and was kept unchanged between exclusion criteria A and B.

Accordingly, under exclusion criterion A, patches were excluded if any of the following conditions were met: (1) the proportion of pixels with a green-channel intensity > 250 exceeded 0.05; (2) the proportion of pixels with a red-channel intensity < 50 exceeded 0.10; or (3) the Tenengrad focus measure was <5000.

Exclusion criterion B was introduced after qualitative visual inspection of Facility B images showed that criterion A excluded lesion-containing regions that remained visually interpretable. Only the color-based halation and blackout rule was modified; the Tenengrad criterion was kept unchanged. Under criterion B, patches were excluded when the proportion of pixels with V > 250 in the hue–saturation–value (HSV) color space exceeded 0.30 or when the proportion with V < 50 exceeded 0.10. These thresholds were selected qualitatively by visual inspection of Facility B images and were not determined by grid search or quantitative optimization. Criterion B was subsequently applied to both Facility A and Facility B datasets for comparison. Thus, criterion B represents a target-site-informed preprocessing adjustment rather than an automatically calibrated or universally prespecified harmonization procedure.

### 2.4. Patch Labeling and Saving

Patches with >95% of their area covered by lesion mask pixels were labeled as lesion patches and saved in a class-specific directory. Patches with <1% lesion coverage were labeled as background patches and saved separately. Each patch was annotated using a rectangular overlay for visualization and review purposes. The patches that did not meet these criteria were excluded.

### 2.5. Model Architecture and Initialization

We adopted a pretrained ResNet50 architecture [17] implemented with Torchvision 0.22.1 in PyTorch 2.7.1 [18]. The final fully connected layer was replaced with a linear layer containing five output units: one background class and four histologic lesion classes (LG, HG, SM1, and SM2). The network was initialized using ResNet50_Weights.DEFAULT, corresponding to ImageNet-pretrained weights [19], and trained end-to-end using the Adam optimizer. Standard ImageNet input normalization associated with the pretrained weights was applied identically to Facility A and Facility B data. No separate cross-site color or intensity harmonization experiment was performed.

#### 2.5.1. Loss Function and Class Balancing

To mitigate class imbalance, class weights were computed as the inverse of the sample counts in each class and normalized to the sum of one. These weights were passed to a cross-entropy loss function.

#### 2.5.2. Training Protocol

For each Facility B fold, the model was trained for a prespecified four epochs with a batch size of 64 using Adam. The learning rate was 1 × 10^−4^ for the first three epochs and was reduced to 1 × 10^−5^ for the fourth epoch. The four-epoch duration was fixed a priori for the revised analysis based on preliminary 20-epoch training experiments in which performance had approximately plateaued by epoch 4. No epoch selection or early stopping was performed using the outer validation partitions. Fold-specific training times and sample sizes are provided in Supplementary Table S1.

#### 2.5.3. Validation Design and Performance Metrics

The estimand of the present study was patch-level classification performance. Lesion identity was not treated as a separate performance evaluation unit. For Facility B site-specific model development, lesion identity was used as the grouping variable for cross-validation to prevent patches derived from the same lesion, including overlapping or near-overlapping patches, from being distributed across the training and validation partitions. Details of the Facility B lesion-grouped cross-validation procedure are provided in Section 2.7.

For cross-institutional evaluation, each of the three Facility A fold-specific models was applied to the same corrected criterion B Facility B cohort. This therefore represented three source models evaluated on one external cohort, rather than three independent external validation cohorts. A five-class confusion matrix was obtained for each Facility A model, and the three confusion matrices were averaged element-wise. Aggregate and class-wise external performance metrics were calculated from this mean confusion matrix.

Overall performance measures included accuracy, macro-precision, macro-F1, balanced accuracy, and macro-specificity. Balanced accuracy was defined as the unweighted mean of class-wise recall, and macro-specificity as the unweighted mean of one-vs-rest class-specific specificities. Class-wise precision and recall are presented with the confusion matrices in the main manuscript, whereas additional patch-level specificity, F1, one-vs-rest ROC-AUC, and AUPRC values are provided in Supplementary Table S2.

Because patches derived from the same lesion are correlated, individual patch counts were not interpreted as independent observations for uncertainty estimation. Historical Facility A internal validation was performed using the original patch-level cross-validation design and is therefore retained only as a descriptive historical result; it was not used as a directly comparable benchmark for the revised lesion-grouped Facility B analysis.

#### 2.5.4. Output and Logging

For each fold, the following outputs were saved: trained model weights (model_foldX.pth), prediction results with softmax probabilities for each class (CSV), confusion matrices (PNG and CSV), and elapsed training time (CSV).

### 2.6. Quantification of Domain Shift Through the Maximum Mean Discrepancy (MMD)

The class-wise maximum mean discrepancy (MMD) [20] was calculated separately for exclusion criteria A and B to quantify residual feature distribution differences between Facilities A and B. For each exclusion criterion, 2048-dimensional feature vectors were extracted from the penultimate layer of the ResNet50 models. To reduce dependence on an arbitrarily selected checkpoint, all three Facility A fold-specific models were used. For fold i, features from the corresponding Facility A held-out partition (A-Pi) and from the complete corrected Facility B dataset for the same exclusion criterion were extracted using the same fold-specific Facility A model. Feature vectors generated by different models were not pooled, concatenated, or averaged.

For each class and fold, equal numbers of Facility A and Facility B patches were sampled. The number of patches sampled from each facility was defined as:

n = min(N_a_, N_b_, 20,000)

where N_A and N_B denote the numbers of available patches for that class in the corresponding Facility A and Facility B datasets, respectively. Sampling was performed without replacement and repeated 10 times using random seeds 0–9.

For each repeat, the Gaussian radial-basis-function (RBF) kernel bandwidth was determined using an exact median heuristic. The sampled Facility A and Facility B feature vectors were combined, and d_med was defined as the median Euclidean distance across all distinct pairs of feature vectors:

d_me_d = median(_a_ < _b_) ||z_a_ − z_b_||_2_

The RBF kernel parameter was then defined as:

γ = 1/(2d_me_d^2^)

and the kernel was defined as:

k(x, y) = exp[−γ||x − y||_2_^2^]

Domain discrepancy was quantified using the biased empirical squared maximum mean discrepancy (MMD^2^):

$${\mathrm{MMD}}^{2}=\frac{1}{n^{2}}\sum_{a=1}^{n}\sum_{b=1}^{n}k\left(x_{a},x_{b}\right)+\frac{1}{n^{2}}\sum_{a=1}^{n}\sum_{b=1}^{n}k\left(y_{a},y_{b}\right)-\frac{2}{n^{2}}\sum_{a=1}^{n}\sum_{b=1}^{n}k\left(x_{a},y_{b}\right)$$

For each random seed, the three fold-specific MMD^2^ estimates were averaged. The reported class-specific MMD^2^ was the mean of these 10 fold-averaged estimates. The standard error was calculated as the standard deviation of the 10 fold-averaged estimates divided by the square root of 10, and the 95% confidence interval was calculated as the mean ± 1.96 standard errors. These intervals quantify variability arising from the repeated patch subsampling procedure after averaging across the three fold-specific feature extractors and should not be interpreted as lesion- or patient-level confidence intervals.

### 2.7. Facility B Site-Specific Model Development

**Figure 2:**
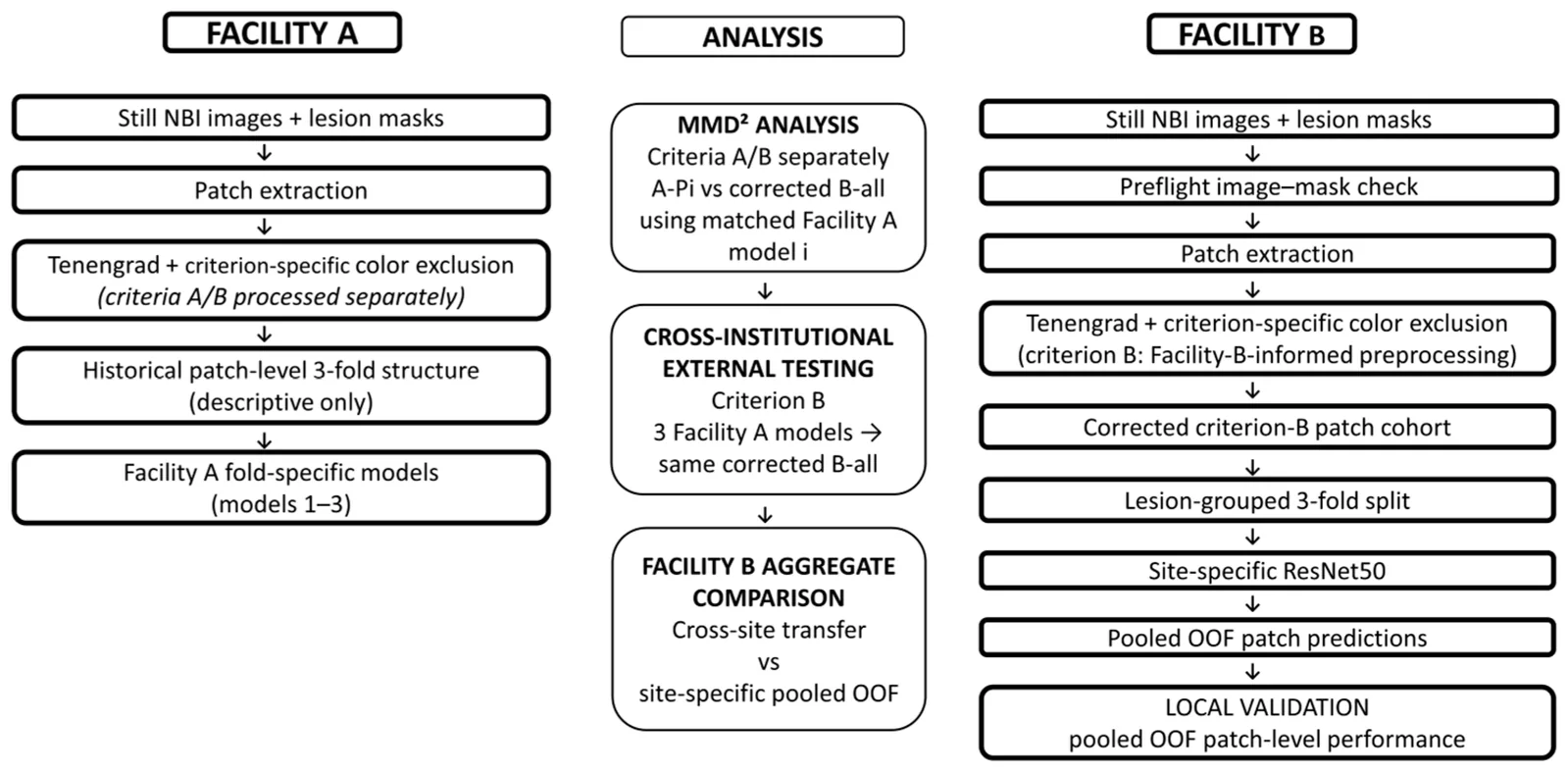
Overview of the revised experimental workflow. Facility A’s historical internal validation is shown descriptively. Cross-institutional evaluation and class-wise MMD^2^ analysis were performed using the corresponding Facility A fold-specific models and corrected Facility B datasets. Facility B site-specific model development used lesion-grouped three-fold cross-validation, and final patch-level performance was calculated from pooled out-of-fold predictions.

Facility B site-specific models were initialized from ImageNet-pretrained ResNet50 weights and fully fine-tuned using the corrected criterion B Facility B dataset. Three-fold cross-validation was performed with lesion-level grouping: each fold contained 268 training lesions and 134 validation lesions, with zero lesion overlap between the two partitions. The numbers of training/validation patches were 94,881/47,997, 98,233/44,645, and 92,642/50,236 for folds 1–3, respectively. The outer validation partitions were used only to obtain out-of-fold predictions and were not used for epoch selection. Final performance was evaluated by pooling the validation predictions from all three folds. This analysis estimates local patch-level performance under lesion-grouped internal validation; it does not demonstrate cross-institutional generalizability of the Facility B models. The overall revised experimental workflow is summarized in Figure 2.

## 3. Results

### 3.1. Input Linkage Audit and Impact of Exclusion Criteria

A preflight audit of the Facility B patch generation pipeline identified two upstream image–mask linkage omissions: one HG image whose mask had not entered the analysis pipeline and one SM2 image whose mask failed filename matching because of a source-filename suffix. Correcting these omissions changed the eligible patch cohort but did not change the predefined patch extraction or quality exclusion criteria, or the 402 source lesions and 469 still images in Facility B (Table 1). Under criterion A, the corrected Facility B dataset contained 39,859 lesion patches and 74,230 background patches (114,089 patches in total). Under criterion B, it contained 50,064 lesion patches and 92,814 background patches (142,878 patches in total).

As shown in Table 2, criterion B increased retained Facility B lesion patches from 12,992 to 16,497 for LG, 14,922 to 19,126 for HG, 7089 to 8207 for SM1, and 4856 to 6234 for SM2; background patch counts also increased from 74,230 to 92,814 in aggregate. Facility A showed only modest changes between criteria A and B. Thus, the Facility-B-informed criterion B materially increased patch retention at Facility B, but this change in the eligible patch cohort should not be interpreted as evidence that the underlying cross-institutional domain shift was resolved.

The image–mask linkage correction and the subsequent lesion-grouped validation reanalysis addressed distinct methodological issues: the former corrected the eligible input patch cohort, whereas the latter changed the Facility B validation design to prevent patches from the same lesion from being distributed across training and validation partitions.

Criterion B was selected qualitatively after visual inspection of Facility B images showed that criterion A excluded lesion-containing regions that remained clinically interpretable. The HSV-based thresholds were not determined by grid search or quantitative optimization and were subsequently applied to both Facility A and Facility B. The Tenengrad focus criterion was kept unchanged between criteria A and B. Accordingly, criterion B should be regarded as a target-site-informed preprocessing adjustment rather than an automatically calibrated or universally prespecified harmonization procedure.

These findings indicate that patch selection rules and upstream image–mask linkage can materially alter the effective patch distribution while leaving the source lesion and still-image cohort unchanged. Both therefore require explicit documentation when cross-institutional model transfer is evaluated.

### 3.2. MMD^2^ Quantification

Using all three Facility A fold-specific feature extractors, the biased empirical squared maximum mean discrepancy (MMD^2^) remained nonzero for every class after the corrected Facility B cohort was used (Table 3). Under criterion B, the mean MMD^2^ values were 0.046659 for BG, 0.095494 for LG, 0.047609 for HG, 0.184937 for SM1, and 0.146650 for SM2. The largest residual feature distribution differences therefore remained in the invasive SM1 and SM2 classes.

Criterion B modestly reduced the mean MMD^2^ for LG (0.098860 to 0.095494), HG (0.054217 to 0.047609), SM1 (0.188065 to 0.184937), and SM2 (0.151421 to 0.146650) relative to criterion A, whereas BG changed minimally (0.046205 to 0.046659). Thus, the Facility-B-informed patch selection adjustment altered the effective data distribution but did not eliminate the inter-institutional feature distribution differences.

Taken together, correction of the Facility B input cohort and application of criterion B were insufficient to eliminate the persistent cross-institutional domain shift quantified in the Facility A feature spaces.

### 3.3. Model Performance

#### 3.3.1. Historical Internal Validation (Facility A)

Historical Facility A internal validation showed high patch-level precision and recall (Table 4A). Re-examination of the original training code confirmed that the three folds were created at the patch level. Because 128 × 128 patches were extracted with a stride of 32 pixels, overlapping or near-overlapping patches from the same lesion could have been distributed across training and validation sets. These historical Facility A values are therefore retained only as descriptive results and are not used as a directly comparable benchmark for the revised lesion-grouped Facility B analysis or as a quantitative measure of cross-site performance loss or subsequent recovery.

#### 3.3.2. External Testing on the Corrected Facility B Cohort

Each of the three Facility A fold-specific models was applied to the same corrected criterion B Facility B cohort of 142,878 patches. A five-class confusion matrix was obtained for each source model, and the three matrices were averaged element-wise to summarize cross-site transfer performance (Table 4B). Thus, this analysis represents three source models evaluated on one external cohort, not three independent external validation cohorts. Aggregate patch-level accuracy was 0.711, macro-precision was 0.413, macro-F1 was 0.397, balanced accuracy was 0.422, and macro-specificity was 0.928. Class-wise recall was 0.896 for BG, 0.204 for LG, 0.675 for HG, 0.036 for SM1, and 0.301 for SM2, demonstrating substantial and class-dependent degradation after cross-institutional transfer.

#### 3.3.3. Lesion-Grouped Site-Specific Model Development in Facility B

Facility B site-specific ResNet50 models were initialized from ImageNet-pretrained weights rather than from the Facility-A-trained checkpoints and were fully fine-tuned under lesion-grouped three-fold cross-validation using the corrected criterion B Facility B dataset. Each fold contained 268 training lesions and 134 validation lesions with zero lesion overlap. Training followed the prespecified fixed four-epoch protocol without validation-based epoch selection or early stopping; fold-specific training times were approximately 26.1–27.4 min. Final performance was calculated from pooled out-of-fold patch predictions, with every lesion contributing to validation predictions in one fold only; fold-specific training and validation patch counts are provided in Supplementary Table S1.

The lesion-grouped Facility B site-specific analysis achieved an accuracy of 0.770, macro-precision of 0.497, macro-F1 of 0.472, balanced accuracy of 0.473, and macro-specificity of 0.936. Class-wise recall was 0.957 for BG, 0.654 for LG, 0.417 for HG, 0.175 for SM1, and 0.160 for SM2 (Table 5). Relative to cross-site application of the Facility A models, recall improved for BG (0.896 to 0.957), LG (0.204 to 0.654), and SM1 (0.036 to 0.175), but decreased for HG (0.675 to 0.417) and SM2 (0.301 to 0.160). Thus, the aggregate improvement was accompanied by substantial class-specific heterogeneity.

#### 3.3.4. Aggregate Comparison on Facility B

On the common Facility B evaluation cohort, site-specific development improved all five aggregate metrics shown in Table 6: accuracy increased from 0.711 to 0.770, macro-precision from 0.413 to 0.497, macro-F1 from 0.397 to 0.472, balanced accuracy from 0.422 to 0.473, and macro-specificity from 0.928 to 0.936. However, the opposing class-wise recall changes in Table 4B and Table 5 demonstrate that this aggregate improvement did not represent uniform superiority across histological classes.

## 4. Discussion

In this study, we re-evaluated persistent cross-institutional domain shift in patch-based NBI classification after correcting two upstream Facility B image–mask linkage omissions and revising the Facility B validation design. Four findings are central. First, the Facility-B-informed criterion B substantially increased patch retention but did not eliminate residual feature distribution differences. Second, MMD^2^ calculated with all three Facility A fold-specific feature extractors remained largest for the invasive SM1 and SM2 classes. Third, all three Facility A source models showed substantial and class-dependent degradation when evaluated on the same corrected criterion B Facility B cohort. Fourth, lesion-grouped site-specific model development at Facility B improved aggregate patch-level metrics, but class-wise effects were heterogeneous, and improved cross-institutional generalizability was not established.

Patch selection and input linkage procedures materially influenced the effective dataset composition. The preflight audit corrected two Facility B linkage omissions without changing the source lesion or still-image cohort. In addition, although criterion A retained the original RGB-based blackout and halation rules, the present study operationally replaced the high-pass-filter focus assessment used in the previous Facility A study [1] with a Tenengrad criterion for computational efficiency [15,16]. The same Tenengrad criterion was applied to both facilities and was unchanged between criteria A and B, whereas criterion B modified only the color-based rule. Criterion B was selected qualitatively after inspection of Facility B images, without grid search or quantitative optimization, and was subsequently applied to both facilities. Thus, it represents a target-site-informed preprocessing adjustment rather than a universally prespecified harmonization method. The unchanged source Facility A cohort should therefore not be taken to mean that the present patch processing pipeline was entirely identical to that of the previous study.

These observations are consistent with prior work showing that domain shifts in medical imaging can arise from differences in acquisition, equipment, and data processing procedures [8,11,21]. Generalizability therefore depends not only on model architecture and dataset size but also on upstream data linkage, curation, quality control rules, and acquisition practices. Standard ImageNet normalization was applied identically to both facilities in the present analysis, but no separate cross-site color or intensity harmonization experiment was performed.

Although the same principal endoscope models and video endoscopy system were available at both facilities, identical hardware does not ensure identical image distributions. Table 1 shows a marked difference in acquisition composition: highly magnified images accounted for 21.1% of Facility A images but only 0.8% of Facility B images. Differences in acquisition practice, lighting, white balance, magnification, image selection, and other site-specific factors are therefore plausible contributors to the observed shift, although the present study does not experimentally isolate their individual effects [11,13,14].

The revised MMD^2^ analysis used all three Facility A fold-specific feature extractors, with each extractor applied to its corresponding Facility A held-out partition and the corrected Facility B dataset for the same exclusion criterion. Criterion B modestly reduced MMD^2^ for most lesion classes, but substantial residual discrepancies remained, particularly for SM1 and SM2. The reported MMD^2^ confidence intervals quantify variability from repeated balanced patch subsampling after averaging across the three feature extractors and are not lesion- or patient-level uncertainty intervals. These findings are consistent with broader evidence that dataset shift can compromise medical image model transfer [7,8,9,10,11,12,22]. Alternative approaches such as CycleGAN-based style transfer [14,23], domain-adversarial learning [24], correlation alignment [25], and feature alignment methods [26] remain important comparators for future work; the present study does not establish superiority of site-specific full fine-tuning over these methods.

The high historical internal performance of Facility A requires cautious interpretation. The original Facility A folds were patch-based, so overlapping or near-overlapping patches from the same lesion could have crossed training and validation partitions, and the present analysis also used an operational Tenengrad focus criterion rather than the high-pass-filter focus assessment described in the previous report. Historical Facility A performance was therefore retained only descriptively and was not used to estimate the magnitude of cross-site performance loss or performance recovery. In contrast, the revised Facility B analysis explicitly prevented patches from the same lesion from crossing training and validation partitions, directly addressing the most immediate leakage concern associated with overlapping patch extraction.

Cross-site evaluation nevertheless demonstrated limited transferability. Each of the three Facility A fold-specific source models was tested on the same corrected criterion B Facility B cohort, so the resulting three confusion matrices represent model-to-model variability on one external dataset rather than three independent external cohorts. Their element-wise mean yielded an accuracy of 0.711 and a balanced accuracy of 0.422, with particularly low recall for LG (0.204) and SM1 (0.036). Such class-dependent degradation, despite corrected input linkage and a common criterion B patch selection rule, reinforces the need for explicit target site evaluation before clinical deployment [8,9,12,27,28].

Facility B site-specific models were developed from the common ImageNet-pretrained ResNet50 initialization rather than by continuing training from Facility A checkpoints. Under lesion-grouped three-fold cross-validation, a prespecified fixed four-epoch protocol required approximately 26.1–27.4 min per fold and yielded a pooled out-of-fold patch-level accuracy of 0.770, macro-precision of 0.497, macro-F1 of 0.472, balanced accuracy of 0.473, and macro-specificity of 0.936. Relative to cross-site transfer, recall increased for BG, LG, and SM1 but decreased for HG and SM2. The reductions for HG and SM2 are clinically important and show why aggregate improvement cannot be interpreted as uniform class-specific superiority. Moreover, because these estimates arise from lesion-grouped internal validation within Facility B, they do not demonstrate external generalizability of the Facility B models. Accordingly, site-specific model development should be interpreted as partially managing the local performance impact of persistent domain shift rather than restoring performance across all classes or eliminating the underlying shift.

Where local annotation is feasible, site-specific model development may therefore be a pragmatic component of deployment when source model transferability is limited. Transfer learning can support efficient local model development [29], while distributed or federated learning may offer complementary strategies when direct data sharing is constrained [30,31]. Prospective multicenter evaluation, however, remains necessary before such approaches can be regarded as clinically generalizable, and local adaptation does not remove the need for local validation.

### Limitations and Future Directions

Several limitations remain. First, the study included only two institutions. Cross-site performance was evaluated on a single corrected Facility B cohort using three Facility A source models, not on three independent external cohorts, and Facility B site-specific performance was assessed by lesion-grouped internal cross-validation rather than by an independent temporal or external target site test set; therefore, improved cross-institutional generalizability was not demonstrated. Second, the performance estimand was the patch. Lesion identity was used as the grouping variable to prevent training–validation leakage, but lesion- or patient-level diagnostic performance was not evaluated; the revised split was lesion-grouped rather than explicitly patient-grouped, and the large number of overlapping patches should not be interpreted as an equivalent number of independent clinical observations. Third, SM1 and SM2 comprised only eleven and eight Facility B lesions, respectively, and the low and heterogeneous class-wise recall underscores uncertainty regarding minority class robustness. Fourth, the MMD^2^ confidence intervals reflect repeated patch subsampling variability after averaging across feature extractors and are not lesion- or patient-level intervals; future performance analyses should incorporate cluster-aware uncertainty estimation. Fifth, the study used ResNet50 and did not experimentally compare modern backbones such as Vision Transformer or ConvNeXt, advanced domain-adaptation or test-time-adaptation methods, additional public or third-site NBI datasets, or deliberate cross-site color/intensity harmonization. Standard ImageNet normalization was applied identically to both facilities, but no separate normalization experiment was performed. Sixth, criterion B was selected qualitatively after visual inspection of Facility B images rather than by an automated calibration procedure, which may limit portability to new sites. Future work should incorporate independent multicenter testing, clinically appropriate lesion- and patient-level endpoints, cluster-aware uncertainty estimation, automated image quality calibration, modern architectures, and direct comparisons with domain adaptation and normalization approaches.

## 5. Conclusions

Correction of two upstream Facility B image–mask linkage omissions and application of the Facility-B-informed criterion B increased the eligible Facility B patch cohort but did not eliminate residual cross-institutional feature distribution differences or the class-dependent degradation of the Facility A models on Facility B. Lesion-grouped Facility B site-specific model development from ImageNet-pretrained weights partially improved aggregate pooled out-of-fold patch-level performance, while recall improved for BG, LG, and SM1 but decreased for HG and SM2. These findings support site-specific model development as a pragmatic strategy for partially managing the local performance impact of persistent domain shift when cross-site transferability is limited, but they do not establish elimination of domain shift or universal cross-institutional generalizability.

## Figures and Tables

**Table 1:** Preparation of endoscopic images and collection of NBI images for the dataset.

A. Facility A						
				**Magnification (Picture Counts/%)**		
Histology	Number of lesions	Number of still pictures	Averaged picture number per lesion	None	Low	High
LG	53.0	294.0	5.5	126/42.9	120/40.8	48/16.3
HG	120.0	840.0	7.0	345/41.1	305/36.3	190/22.6
SM1	20.0	138.0	6.9	59/42.8	46/33.3	33/23.9
SM2	17.0	118.0	6.9	40/33.9	56/47.5	22/18.6
total	210.0	1390.0	6.6	570/41.0	527/37.9	293/21.1
B. Facility B						
				**Magnification (Picture Counts/%)**		
Histology	Number of lesions	Number of still pictures	Averaged picture number per lesion	None	Low	High
LG	257.0	257.0	1.0	175/68.1	82/31.9	0/0
HG	126.0	126.0	1.0	78/61.9	48/38.1	0/0
SM1	11.0	50.0	4.5	32/64.0	14/28.0	4/8.0
SM2	8.0	36.0	4.5	23/63.9	13/36.1	0/0
total	402.0	469.0	1.2	315/65.8	160/33.4	4/0.8

NBI, narrow-band imaging; HG, high-grade dysplasia; LG, low-grade dysplasia; SM1, superficially invasive submucosal carcinoma; SM2, deeply invasive submucosal carcinoma.

**Table 2:** Impact of exclusion criteria on dataset construction between institutions.

A. Facility A		
Exclusion criteria A		
Class	Lesion patch count	Background patch count
LG	55,185	28,052
HG	177,096	68,590
SM1	30,125	17,218
SM2	22,705	9068
Exclusion criteria B		
Class	Lesion patch count	Background patch count
LG	59,274	31,314
HG	189,620	76,473
SM1	31,658	18,676
SM2	23,329	9886
B. Facility B		
Exclusion criteria A		
Class	Lesion patch count	Background patch count
LG	12,992	49,644
HG	14,922	16,802
SM1	7089	4369
SM2	4856	3415
Total	39,859	74,230
Exclusion criteria B		
Class	Lesion patch count	Background patch count
LG	16,497	61,518
HG	19,126	21,477
SM1	8207	5303
SM2	6234	4516
Total	50,064	92,814

HG, high-grade dysplasia; LG, low-grade dysplasia; SM1, superficially invasive submucosal carcinoma; SM2, deeply invasive submucosal carcinoma. Facility B values incorporate the preflight correction of two image–mask linkage omissions; source lesion and still-image counts were unchanged.

**Table 3:** Quantification of residual domain shift using MMD^2^.

Exclusion criteria A						
Class	Samples (F1/F2/F3)	Fold 1 MMD^2^	Fold 2 MMD^2^	Fold 3 MMD^2^	MMD^2^ mean	95% CI
BG	20,000/20,000/20,000	0.045204	0.044231	0.049181	0.046205	[0.046075, 0.046335]
LG	12,992/12,992/12,992	0.100428	0.092941	0.103212	0.098860	[0.098679, 0.099042]
HG	14,922/14,922/14,922	0.059239	0.054537	0.048875	0.054217	[0.054087, 0.054347]
SM1	7089/7089/7089	0.196592	0.186821	0.180780	0.188065	[0.187813, 0.188316]
SM2	4856/4856/4856	0.160398	0.150832	0.143034	0.151421	[0.151080, 0.151762]
Exclusion criteria B						
Class	Samples (F1/F2/F3)	Fold 1 MMD^2^	Fold 2 MMD^2^	Fold 3 MMD^2^	MMD^2^ mean	95% CI
BG	20,000/20,000/20,000	0.045166	0.045910	0.048902	0.046659	[0.046487, 0.046832]
LG	16,497/16,497/16,497	0.100414	0.091735	0.094333	0.095494	[0.095342, 0.095645]
HG	19,126/19,126/19,126	0.047991	0.046536	0.048299	0.047609	[0.047467, 0.047750]
SM1	8207/8207/8207	0.202319	0.177891	0.174600	0.184937	[0.184793, 0.185081]
SM2	6234/6234/6234	0.132626	0.138180	0.169143	0.146650	[0.146422, 0.146877]

MMD^2^, squared maximum mean discrepancy; CI, confidence interval; BG, background; HG, high-grade dysplasia; LG, low-grade dysplasia; SM1, superficially invasive submucosal carcinoma; SM2, deeply invasive submucosal carcinoma. MMD^2^ values were calculated independently with the three Facility A fold-specific feature extractors and aggregated at the scalar estimate level. The reported 95% CI reflects variability across 10 repeated patch subsampling runs after averaging across the three feature extractors and should not be interpreted as lesion- or patient-level uncertainty.

**Table 4:** Historical Facility A internal performance and cross-site external performance on Facility B.

A. Historical Facility A internal patch-level cross-validation (descriptive).								
	BG	LG	HG	SM1	SM2	Ground truth total	Precision	Recall
BG	105,037	208	389	54	22	105,710	0.99	0.99
LG	145	54,467	524	38	11	55,185	0.95	0.99
HG	1064	2566	171,579	1176	711	177,096	0.99	0.97
SM1	16	17	86	29,991	15	30,125	0.96	1.00
SM2	10	14	118	12	22,551	22,705	0.97	0.99
B. Mean external confusion matrix from three Facility A fold-specific models applied to the identical corrected Facility B cohort.								
Ground truth	BG	LG	HG	SM1	SM2	Ground truth total	Precision	Recall
BG	83,173.7	1500.7	5882.0	1029.0	1228.7	92,814	0.966	0.896
LG	957.0	3365.0	10,220.0	803.0	1152.0	16,497	0.435	0.204
HG	1416.3	1738.0	12,906.7	1395.0	1670.0	19,126	0.345	0.675
SM1	237.0	360.3	5618.3	295.7	1695.7	8207	0.074	0.036
SM2	333.0	766.0	2768.7	492.0	1874.3	6234	0.246	0.301

BG, background; HG, high-grade dysplasia; LG, low-grade dysplasia; SM1, superficially invasive submucosal carcinoma; SM2, deeply invasive submucosal carcinoma. Table 4A shows historical Facility A patch-level cross-validation and is descriptive only. Table 4B shows the mean confusion matrix from three Facility A fold-specific models evaluated on the identical corrected Facility B cohort; therefore, mean cell counts may be non-integers.

**Table 5:** Facility B site-specific pooled out-of-fold patch-level confusion matrix.

Ground Truth	BG	LG	HG	SM1	SM2	Ground Truth Total	Precision	Recall
BG	88,819	1705	1519	350	421	92,814	0.947	0.957
LG	1035	10,797	3813	558	294	16,497	0.470	0.654
HG	2590	6611	7979	997	949	19,126	0.432	0.417
SM1	825	2487	2573	1435	887	8207	0.355	0.175
SM2	544	1392	2597	701	1000	6234	0.282	0.160

BG, background; HG, high-grade dysplasia; LG, low-grade dysplasia; SM1, superficially invasive submucosal carcinoma; SM2, deeply invasive submucosal carcinoma. Performance was calculated from pooled out-of-fold patch predictions under lesion-grouped three-fold cross-validation.

**Table 6:** Aggregate patch-level performance comparison on Facility B.

Evaluation on Facility B	Accuracy	Macro-Precision	Macro-F1	Balanced Accuracy	Macro-Specificity
Facility A models -> Facility B external	0.711	0.413	0.397	0.422	0.928
Facility B site-specific pooled OOF	0.770	0.497	0.472	0.473	0.936

Note: The external row summarizes three Facility A fold-specific models evaluated on the identical corrected Facility B cohort. The site-specific row summarizes lesion-grouped, three-fold pooled out-of-fold predictions. Balanced accuracy is equivalent to macro-recall and is therefore not duplicated.

## Data Availability

The data generated or analyzed during this study are included in this article and its Supplementary Materials. Some underlying datasets are not publicly available because of privacy restrictions, but can be obtained from the corresponding author upon reasonable request.

## Supplementary Materials

The following supporting information can be downloaded at: https://www.mdpi.com/article/10.3390/cancers18183002/s1, Table S1: Facility B lesion-grouped training process; Table S2: Detailed Facility B pooled out-of-fold patch-level performance.
