# Supplementary material for: Persistent Cross-Institutional Domain Shift in Narrow-Band Imaging Classification of Colorectal Lesions and the Utility of Site-Specific Fine-Tuning
Source: Cancers (Basel). 2026 Sep 16;18(18):3002. doi: 10.3390/cancers18183002 (PMC13604798; doi:10.3390/cancers18183002)
Supplement: Supplementary file 1 [file cancers-18-03002-s001.zip › cancers-4516570-supplementary.pdf]

Supplementary Tables

Persistent Cross-Institutional Domain Shift in Narrow-Band Imaging Classification of Colorectal Lesions and the Utility of Site-Specific Fine-Tuning

Table S1. Facility B lesion-grouped training process.

Fold	Fixed epochs	Training lesions	Validation lesions	Training patches	Validation patches	Training time
1	4	268	134	94,881	47,997	26.37 min
2	4	268	134	98,233	44,645	27.40 min
3	4	268	134	92,642	50,236	26.09 min

All folds used lesion-grouped three-fold cross-validation. All patches from a lesion were confined to one partition within a fold, and the outer validation partitions were not used for epoch selection or early stopping. Final performance was calculated from pooled out-of-fold patch predictions.

Table S2. Detailed Facility B pooled out-of-fold patch-level performance.

Class	Support	Precision	Sensitivity / Recall	Specificity	F1	ROC-AUC	AUPRC
BG	92,814	0.947	0.957	0.900	0.952	0.980	0.988
LG	16,497	0.470	0.654	0.904	0.547	0.889	0.573
HG	19,126	0.432	0.417	0.915	0.424	0.818	0.393
SM1	8,207	0.355	0.175	0.981	0.234	0.746	0.212
SM2	6,234	0.282	0.160	0.981	0.204	0.734	0.159

BG, background; LG, low-grade dysplasia; HG, high-grade dysplasia; SM1, superficially invasive submucosal carcinoma; SM2, deeply invasive submucosal carcinoma. Metrics were calculated from pooled out-of-fold patch predictions under lesion-grouped three-fold cross-validation. The performance evaluation unit was the patch; lesion identity was used only as the grouping variable for data splitting. Confidence intervals are not shown because overlapping patches are correlated and naive patch-wise intervals would overstate precision.
